# Total knee arthroplasty in pigmented villonodular synovitis osteoarthritis: a systematic review of literature

**DOI:** 10.1007/s12306-023-00793-y

**Published:** 2023-06-20

**Authors:** A. Panciera, A. Colangelo, A. Di Martino, R. Ferri, B. D. Bulzacki Bogucki, D. Cecchin, M. Brunello, L. Benvenuti, V. Digennaro

**Affiliations:** 1St Orthopaedic and Traumatologic Clinic, IRCCS Istituto Ortopedico Rizzoli, Via G.B. Pupilli 1, 40136 Bologna, Italy

**Keywords:** Total knee arthroplasty, Pigmented villonodular synovitis, Outcome, Review

## Abstract

**Purpose:**

Pigmented Villonodular Synovitis (PVNS) is a proliferative disease arising from the synovial membrane, mainly affects large joints such as the knee (almost 80% of total). Prostheses implanted in PVNS osteoarthritis show a higher revision rate when compared to primary osteoarthritis, due to the recurrence of disease and the overall surgical complications. The purpose of this systematic review is to summarize and compare indications, clinical and functional outcomes, disease-related and surgical-related complications of total knee arthroplasty in PVNS osteoarthritis.

**Materials and methods:**

A systematic review of the literature was performed with a primary search on Medline through PubMed. The PRISMA 2009 flowchart and checklist were used to edit the review. Screened studies had to provide preoperative diagnosis, previous treatments, main treatment, concomitant strategies, mean follow-up, outcomes and complications to be included in the review.

**Results:**

A total of 8 articles were finally included. Most of papers reported the use of non-constrained design implants, mainly posterior stabilized (PS) and in case of PVNS with extensive joint involvement implants with higher degree of constraint to obtain a fulfilling balancing. Recurrence of PVNS has been indicated as the major complication, followed by aseptic loosening of the implant and difficult post-operative course with an increased risk of stiffness.

**Conclusion:**

Total knee arthroplasty represents a valid treatment for patients with PVNS end-stage osteoarthritis, with good clinical and functional results, even in longer follow-up. It would be advisable a multidisciplinary management and a meticulous rehabilitation and monitoring following the procedure, to reduce the emergence of recurrence and overall complications.

## Introduction

Pigmented Villonodular Synovitis (PVNS), also known as tenosynovial giant cell tumor (TGCT), is a proliferative disease of the histocytes arising from the synovial membrane which can affect joints, tendon sheaths and bursae [1, 2].

PVNS mainly affects large joints such as the knee (almost 80% of total), followed by hip and ankle [3].

It is considered as a benign cell proliferation, and it is classified into a localized-type (L-PVNS) and a diffused-type (D-PVNS). These variants differ in their clinical and radiological presentations but are similar on histology with histocytes proliferation and hemosiderin deposition inside the synovial tissue [4].

The localized form is usually described as a distinct mass within the synovium, opposite to diffuse PVNS that involves the entire synovium, whether it can be intra-articular or extra-articular, and can be locally aggressive with a propensity for local recurrence [5].

Treatment strategies are mainly based on the excision of the pathological tissue, but nowadays the best standard of care remains unclear. Surgical resection is the primary treatment for patients with both localized and diffuse PVNS. This includes arthroscopic and open excision, with partial or extensive synovectomy. [6]

In addition to the surgical resection, a variety of treatments have been used to achieve good results in PVNS, including external beam radiation [7], radiosynoviorthesis (mostly ^90^yttrium) [8–10], cryosurgery [11] and recently targeted therapy with monoclonal antibodies [12–16] and specific inhibitor molecules [16–19].

Owing to the aggressive and relapsing nature of PVNS, despite the attempted treatments, there is often an early onset osteoarthritis and the need for an arthroplasty procedure [20, 21], which is usually performed through an extensive exposure of the joint that ensures a large debridement of the synovial membrane [6]. Even though an arthroplasty procedure allows for satisfying clinical and functional outcomes, prosthesis implanted in PVNS osteoarthritis shows a higher revision rate when compared to primary osteoarthritis especially in diffuse variants, due to recurrence of the disease and overall surgical complications [22].

Literature provides several case-report and case-series describing arthroplasty procedures in end-stage PVNS osteoarthritis but lacks a systematic collection of these works. The purpose of this systematic review is to summarize and compare indications, clinical and functional outcomes, disease-related and surgical-related complications of TKA in end-stage PVNS osteoarthritis.

## Material and methods

A systematic review of the literature was performed with a primary search on Medline through PubMed using the following keywords: ((PVNS) OR (villonodular synovitis) OR (giant cell tumor of tendon sheath) OR (tenosynovial tumor)) AND ((Total knee) OR arthroplasty).

The inclusion criteria were: studies providing clinical and functional results and complications concerning the outcomes of PVNS osteoarthritis treated with knee arthroplasty; retrospective or prospective clinical studies including randomized controlled trials, non-randomized trials, cohort studies, case-control, case-reports and case-series studies; papers in English without any restriction on publication date.

The exclusion criteria were: review articles; in vitro or experimental biomechanical or cadaveric studies; papers not in English; studies concerning isolated PVNS performed without concomitant arthroplasty procedures.

Articles were initially identified based on title and abstract: full-text versions of relevant trials were then obtained and evaluated. References of the identified articles were checked not to miss any further relevant articles. The PRISMA 2020 flowchart and checklist were considered to edit the review.

The Level of Evidence (LOE) of the studies was assigned based on the 2011 Oxford Center for Evidence-based Medicine Levels of Evidence [23].

The following data, when available, were extracted from the articles: year of publication, Level of Evidence, number of treated knees, mean age of patients, preoperative diagnosis, previous treatments, main treatment, concomitant strategies, adjuvant treatments, mean follow-up, clinical and functional outcomes, disease specific and surgical-related complications.

## Results

**Fig. 1:**
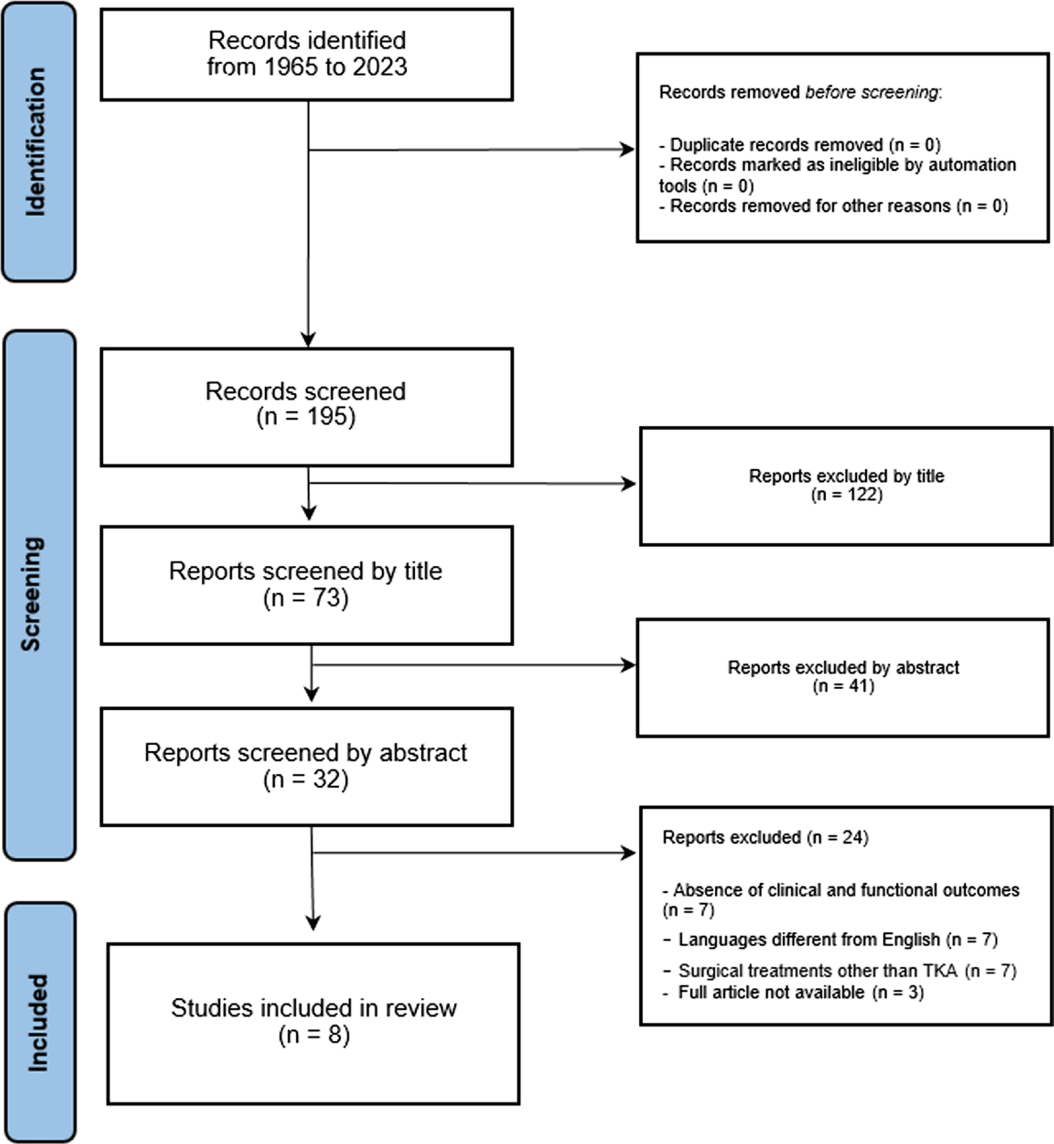
PRISMA flow diagram illustrates the studies that have been identified, included, and excluded

A total of 8 articles were finally included in the systematic review. The PRISMA diagram illustrates the studies that have been identified, included, and excluded (Fig. 1).

Table 1 describes data extracted from the included papers.

**Table 1:** **S**ummary of the data extracted from the included studies, presented in a chronological order based on the publication dates

References	LOE	N knees	Mean Age	Preoperatively diagnosis	Previous treatment	Mean treatment
Hamlin et al. [20]	VI	18 patients	IA: 59.3 (40–82) IB: 64.3 (60–73) II: 63.3 (56–73)	PVNS IA: active diffuse (11 patients) IB: inactive diffuse (3 patients) II: focal (4 patients)	IA: 8 synovectomies (5 arthroscopies) 1 external beam irradiation IB: 3 synovectomies (1 patient twice) 1 external beam irradiation II: No previous treatment	IA: 10 TKA (8 cemented, 1 Hybrid fixation, 1 porous ingrowth) 1 UKA (cemented) IB: 3 TKA (3 cemented) II: 4 TKA (4 cemented)
Verspoor et al. [2]	IV	12	55.8 (33–73)	PVNS–8 diffuse, 4 focal	Patients A: 2 synovectomies, 1 partial meniscectomy, 1 2-stage synovectomy, 1 Cryotherapy and 1 external beam radiation therapy B: 1 nodulectomy C: no previous treatments for PVNS D: 1 synovectomy, 1 2-stage synovectomy E: 2 synovectomy F: no previous treatments for PVNS G: 1 synovectomy H: no previous treatments for PVNS I: 1 2-stage synovectomy, 1 Yttrium radiosynovectomy J: no previous treatments for PVN K: 1 synovectomy, 1 Yttrium radiosynovectomy L: 3 synovectomies, 1 Yttrium radiosynovectomy, 1 Imatinib therapy	TKA 7 PS 2 CR 2 PFA 1 HP
Pinheiro J. et al. [3]	V	1	65	PVNS–Diffuse type	1 Arthroscopic synovectomy 1 open synovectomy 1 external beam irradiation	TKA 1 PS
Lei et al. [21]	IV	11	61.7 (50–70)	PVNS–7 diffuse, 4 focal	No previous treatment	TKA 11 PS
Houdek et al. [24]	III	48	61 (36–94)	PVNS–40 diffuse, 8 focal	4 posterior synovectomies 3 external beam irradiation	TKA 28 PS 14 CR 2 semi-constrained 2 rotating hinges 2 UKA
Lin et al. [25]	III	17	58.6 (51.4–65.8)	PVNS–diffuse type	7 patients underwent at least one synovectomy	TKA 11 CR
Su et al. [4]	IV	29	61.9 (48–81)	PVNS–diffuse type	4 arthroscopy	TKA 29 PS (cemented)
Abdul-Aziz et al. [26]	V	1	79	PVNS–diffuse type	No previous treatments	TKA: 1 Constrained Hinged

Concomitant strategy	Mean Follow-Up	Clinical and Functional outcomes	PVNS related Complications	Surgery related complications
IA: 11 total synovectomies IB no concomitant strategy II 4 partial synovectomies	10.3 years (3.6–20.1 years)	IA Mean KSS clinical and Functional Scores improved from 48 (27–71) and 43 (0–80) preoperatively to 88 (80–90) and 74 (20–100) post-operatively IB: Mean KSS clinical and Functional Scores improved from 37 (12–70) and 32 (40–50) preoperatively to 91 (90–92) and 72 (60–95) post-operatively II: Mean KSS clinical and Functional Scores improved from 45 (40–50) and 64 (50–80) preoperatively to 89 (83–97) and 86 (75–100) post-operatively	IA 2 recurrences: 1 diagnosed during revision arthroplasty for aseptic loosening of the implant 1 required an above the knee amputation for a severe recurrence with invasion of muscles and Severe Pain Syndrome IB No PVNS related complications II No PVNS related complications	IA 2 manipulation under the anesthesia for persistent flexion limitation 3 had a revision arthroplasty for aseptic loosening of the implant IB No surgery related complications II 1 manipulation under the anesthesia for persistent flexion limitation
A: no concomitant strategies B: no concomitant strategies C: no concomitant strategies D: 1 synovectomy E: 1 2-stage synovectomy, 1 cryotherapy F: 1 synovectomy G: 1 synovectomy H: no concomitant strategies I: no concomitant strategies J: no concomitant strategies K: no concomitant strategies L: 1 synovectomy	5.5 (0.2–13) years	KSS clinical score: 7 patients had excellent results (80–100), 1 good results (70–79) and 3 had poor results (< 60) KSS function knee scores: 6 patients had excellent results (80–100) and the other 5 patients had poor results (< 60)	1 recurrence of PVNS (no intervention declared)	1 manipulation under the anesthesia for persistent flexion limitation 1 revision arthroplasty for aseptic loosening of the implant 1 surgical neurolysis for patient neuropathic pain
1 total synovectomy EV tranexamic acid (in anesthetic induction and 15 min before the releasing of tourniquet)	30 days	The patient had clinical and functional improvement without a specific score system declared	No PVNS related complications	No surgery related complications
4 partial synovectomies in Lt–PVNS 7 total synovectomies for Dt–PVNS	61 months (39–83)	Mean KSS clinical score and KSS function score improved from 40.5 (32.4–48.6) and 35.0 (21.2–48.8) preoperatively to 90.0 (85.9–94.1) and 81.8 (74.3–89.3) post-operatively	No PVNS related complications occurred during the follow-up period	No surgery related complications found at the latest follow-up
10 total synovectomies 27 partial synovectomies of focal areas of PVNS	14 years (2–35)	Mean KSS Clinical and Functional scores improved from 54 (20–77) and 45 (0–90) preoperatively to 87 (37–100) and 62 (0–100) post-operatively	6 recurrences: 4 treated with synovectomy and revision TKA for component loosening, 1 local excision 1 transfemoral amputation	10 revision TKA (4 for PVNS recurrence, 3 for component loosening and osteolysis, 1 tibial component fracture, 1 instability, 1 deep infection) 3 hematomas 2 instability 2 temporary peroneal nerve palsy 2 chronic soft tissue pain 1 patella maltracking 1 patellar clunk
11 total synovectomies	7.2 years (5.5–8.9)	Mean KSS Clinical and Functional scores improved from 36 (32.8–39.2) and 37.9 (35.2–40.6) preoperatively to 93.5 (98.7–97.3) and 88.2 (86.8–89.6) post-operatively	No PVNS related complications occurred during the follow-up period	1 revision TKA for periprosthetic fracture 3 patients had stiffness one year after surgery 1 patient suffered of chronic soft tissue pain
29 total synovectomies	59 months (36–84)	Mean KSS Clinical and Functional scores improved from 38.9 (17–54) and 48.9 (25–80) preoperatively to 84.4 (75–98) and 84.6 (75–95) post-operatively	No PVNS related complications occurred during the follow-up period	No surgery related complications found at the latest follow-up
1 total synovectomy	19 months	The patient had clinical and functional improvement without a specific score system declared	No PVNS related complications occurred during the follow-up period	No surgery related complications found at the latest follow-up

LOE: Level Of Evidence (according with the 2011 Oxford Center for Evidence-based Medicine Levels of Evidence); PVNS: Pigmented Villonodular Synovitis; Lt: Localized type; Dt: Diffuse Type; TKA: Total Knee Arthroplasty; UKA: Unicondylar Knee Arthroplasty; PFA: Patello—Femoral Arthroplasty; HP: hemiarthroplasty of the knee; Double-stage synovectomy: an anterior synovectomy followed by a posterior synovectomy 4–6 weeks later; PS: Posterior Stabilized; CR: Cruciate Retaining; FU: Follow-Up; ROM: Range of Movement; KSS: Knee Society Score

Most of the papers were rated as level IV according to the 2011 Oxford Center for Evidence-based Medicine Levels of Evidence; just two studies were rated as level V being case reports and two studies as level III. All selected studies provide clinical and functional results and complications concerning the management of PVNS treated with total knee arthroplasty.

## Discussion

From the selected studies, the first goal was to highlight the design and degree of constraint of the prosthetic implants adopted by authors in PVNS osteoarthritis. Most of papers reported the use of non-constrained design implant such as posterior stabilized (PS) or cruciate retaining (CR) models, obtaining satisfying outcomes [2–4, 20, 21, 24, 25]. Although these implant designs lead to comparable results in clinical and functional scores improvement, in PVNS osteoarthritis, PS system may be preferable over the CR one, as it allows a more extensive joint exposure, necessary to perform an accurate synovial debridement [4, 20, 21].

In case of PVNS with extensive joint involvement, a massive debridement of the synovium is required, often sacrificing portions of capsule and ligamentous apparatus. Implants with higher degree of constraint are needed here to obtain fulfilling balancing: semi-constrained [24], even up to hinged system have been used in this situations [24, 26]. Authors did not point out different clinical or functional outcomes depending on the degree of constraint of the implants over time: however, only two of the included studies underline a follow-up period longer than 10 years [27].

Furthermore, Hamlin et al. [20] and Houdek et al. [24] reported the use of unicompartmental prothesis in 1 and 2 procedures out of 18 and 48, respectively, without specifying what features of the disease have allowed the authors to undertake a partial arthroplasty procedure.

Regarding the fixation of the prothesis, only two studies have described the system: Su et al. [4] placed cemented implant for the whole 29 patients, while Hamlin et al. [20] selected cemented implants in 16 out of 18 patients, 1 porous ingrowth and 1 hybrid fixation implant, without reporting differences in terms of outcomes among the fixation systems.

Most of patients underwent different therapies before the arthroplasty procedure, including external beam irradiation [2, 3, 20, 24], cryotherapy [2], ^90^Yttrium radiosynovectomy [2] or even a systemic therapy with Imatinib [2]. Unfortunately, the design of those studies did not allow to clarify the specific single impact of these previous treatments on the clinical or functional outcomes of the arthroplasty surgery.

Recurrence of the disease is represented recurrent effusions, hemarthrosis, lytic formation [24], new palpable mass or a worsening pain with or without a range of motion reduction [4]. Recurrence is a time-dependent phenomenon, and longer follow-up may increase its incidence [1]. It is also hardly associated with the extension of the synovectomy performed during the arthroplasty procedure, [1] and some authors suggest an extension of the synovectomy depending on the level of activity and diffusion of the PVNS at the time of surgery. Specifically, Hamlin et al. [20] adopted a total synovectomy in case of active diffuse type disease, a partial synovectomy in case of local disease and isolated arthroplasty without any debridement if the disease comes as inactive status. Similarly, Lei et al. [21] suggest a total synovectomy only for diffuse disease and the adequacy of a partial debridement in the focal showing.

Hamlin et al. reported a recurrence rate of 11% [20] while Houdek et al. about 13% [24], with an average gap of 6 years (range 2–12) from the procedure. In these cases, all patients with the recurrence had a diffuse and active disease at the time of diagnosis [2, 20, 24].

Literature provides different options to cope with the recurrence after the arthroplasty procedure, mainly based on additional synovectomies of the involved tissue. This supplementary debridement could be performed alone if the components appear well-fixed or associated with a revision procedure in case of prosthesis mobilization. Houdek et al. [24] found that two or more previous surgical procedures to remove PVNS tissue were associated with an increased risk of disease recurrence following the arthroplasty. In fact, the effects of iatrogenic surgical trauma could result in a harder identification of the pathological synovial tissue, with a higher risk of leaving parts of it in situ [28].

Moreover, as suggested by Mollon et al. [7], and Hamlin et al. [20], a good strategy to reduce local recurrence of PVNS may consist in the addition of peri-operative radiotherapy, with external beam or intra-articular irradiation, especially if the disease involves the surrounding soft tissues [20].

The choice of appropriate treatments in the recurrences is naturally affected by the extension and symptomatology of the disease. In fact, as described by Verspoor et al. [2], a small or asymptomatic recurrence could be managed conservatively with only observation, especially if *incidentally detected* by instrumental examinations. Rarely, the recurrence of PVNS after arthroplasty procedures could occur as a severe and destructive process requiring massive interventions. Hamlin et al. [20] and Houdek et al. [24] reported one patient each with large recurrence including the invasion of muscles groups and neuro-vascular impairment with severe pain syndrome, treated with above-knee amputation.

In PVNS arthroplasty procedures, authors described the disease-related complications. First of all, patients affected by diffuse PVNS may have a more difficult post-operative course with an increased risk of stiffness if compared to those who performs a TKA for primary osteoarthritis [24, 29]. This is probably due to the disease process, previous intervention, and the arthroplasty procedure itself, which often requires a large synovial debridement to be eventually followed by a larger fibrous reaction. In the included studies, stiffness and persistent flexion limitation were treated successfully by using manipulation under anesthesia, without the need for surgery. Only one patient in Hamlin et al. [20] required a posterior capsular release for persisting stiffness.

Another serious complication frequently highlighted by authors is aseptic loosening of the implant, resulting in prosthetic failure. Hamlin et al. [20] reported 3 revision procedures of the prosthesis out of 18 primary arthroplasty, while Houdek et al. [24] about 10 out of 48. Aside from those with a mean follow-up lower than 5 years, all studies have found at least one patient revised for the loosening of the implant. The underlying mechanism seems twofold: correlations with the inflammatory substrate associated with PVNS and the frequent use of prosthetic implants with a greater degree of constraint. Despite this data, no statistical differences were reported in the revision rate between TKA conducted in PVNS and in primary OA. The same goes for the other reported adverse events, including neuropathic pain [2], chronic soft tissue suffering [21, 24], hematomas and deep infection, temporary peroneal nerve palsy, patella maltracking and patellar clunk. [24]

A meta-analysis of the results could not be performed from this systematic review because of the insufficient statistical power correlated with the low number of cases in some of the included studies and the heterogeneity related to the different clinical and functional score system used by authors to describe their results. Furthermore, two of the included studies have not declared any specific score system and provided only a qualitative description of the results.

A last limitation of this review concerns the use in many included works of combined procedures to manage the PVNS; thus, it could be difficult in certain articles to interpret the relative contribution of single techniques in the final results.

## Conclusions

Prosthetic knee replacement represents a valid treatment for patients with PVNS end-stage osteoarthritis, with good clinical and functional results reported by author, even in longer follow-up. Arthroplasty procedures are frequently performed using prostheses with high degree of constraint, necessary to a wide exposure and an accurate debridement of the pathological tissue. Recurrences are frequent and hardly associated with the extension of the synovectomy, mainly in diffuse forms. Moreover, authors point out an increase in overall surgical complication, first of all a higher risk of stiffness if compared to TKA performed in primary osteoarthritis. Given the intricacy course of PVNS, it would be advisable a multidisciplinary management including a neo-adjuvant treatment in preparation for the arthroplasty and a meticulous rehabilitation and monitoring following the intervention, to reduce the emergence of recurrence and complications over the long term.
